# Supplementary material for: Procyanidins and Anthocyanins in Young and Aged Prokupac Wines: Evaluation of Their Reactivity Toward Salivary Proteins
Source: Foods. 2025 May 17;14(10):1780. doi: 10.3390/foods14101780 (PMC12111099; doi:10.3390/foods14101780)
Supplement: Supplementary file 1 [file foods-14-01780-s001.zip › foods-3619995-supplementary.pdf]

Table S1. Equation parameters of phenolic standards used for quantification.

Compounds	y=ax±b	R2	LOD (µg/mL)	LOQ (µg/mL)
**Coumaric acid**	y = 25243511.3354x - 866412.4214	0.9961	0.38	1.25
**Vanillic acid**	y = 913191.4989x + 31480.0391	0.9985	0.27	0.89
**Gallic acid**	y = 13648904.1369x - 768789.9173	0.9972	0.26	0.88
**Caffeic acid**	y = 59357328.4218x + 6466375.9189	0.9959	0.27	0.91
**Ferulic acid**	y = 6680790.5687x + 216092.6620	0.9977	0.18	0.61
**Epicatechin**	y = 17883934.8558x + 1151944.3976	0.9962	0.16	0.54
**Catechin**	y = 14379602.5826x + 578077.8336	0.996	0.31	1.04
**Kaempferol**	y = 54512598.2797x + 4271269.9233	0.9864	0.17	0.57
**Myricetin**	y = 24260634.2632x + 722991.6082	0.9904	0.43	1.44
**Naringenin**	y = 30822249.0218x + 7852515.7889	0.99	0.32	1.07
**Resveratrol**	y = 7123109.0475x + 1067695.8248	0.9981	0.31	1.03
**Ellagic acid**	y = 13775863.4526x + 578964.5772	0.9966	0.16	0.52
**Procyanidin B2**	y = 12351809.4676x - 258435.3175	0.9988	0.14	0.45
**Malvidin-3-*O*-glucoside**	y = 11838421.7623x + 1430652.8398	0.9963	0.3	1.01

Table S2. Untargeted UHPLC Q-ToF MS phenolic profile of young and aging Prokupac red wine, and ratio of each compound identified in young and aged wine.

No.	RT	Compounds	Formulas	Calculated mass	*m/z* exact mass	mDa	MS fragments (main fragment)	Ratio YPW/APW
				Phenolic acid and derivatives				
**1**	2.80	**Coumaric acid**	$C_9H_7O_3^-$	163.0395	163.0401	0.58	**119.0497(100)**	–
**2**	7.38	**Vanillic acid**	$C_8H_7O_4^-$	167.0344	167.0356	1.17	**123.0439(100)**, 107.0133	–
**3**	1.00	**Gallic acid**	$C_7H_5O_5^-$	169.0137	169.0148	1.10	**125.0239(100)**, 124.0163	**1.2**
**4**	4.37	**Caffeic acid**	$C_9H_7O_4^-$	179.0344	179.0356	1.17	**135.0445(100)**, 134.0371, 107.0499	**1.4**
**5**	3.92	**Ferulic acid**	$C_{10}H_9O_4^-$	193.0501	193.0503	0.22	**134.0365(100)**, 133.0283, 117.0342, 148.0133, 164.0119	–
**6**	6.59	**Ethyl gallate**	$C_9H_9O_5^-$	197.045	197.0465	1.50	**124.0162(100)**, 125.0227, **169.0144**	**1.1**
**7**	9.42	**Ethyl caffeic acid**	$C_{11}H_{11}O_4^-$	207.0657	207.0670	1.27	**133.0292(100)**, 135.0446, 134.036, 161.0244, **179.0343**	**0.25**
**8**	3.20	**Coutaric acid**	$C_{13}H_{11}O_8^-$	295.0454	295.0470	1.61	**119.0501(100)**, **163.0400**	**2.2**
**9**	7.52	**Ellagic acid**	$C_{14}H_5O_8^-$	300.9984	301.0001	1.66	**300.9992(100)**, 299.9913, 283.9966, 229.016, 201.0202, 151.0033, 245.0144, 185.0251, 173.0229, 257.0103	**0.51**
**10**	1.54	**Caftaric acid**	$C_{13}H_{11}O_9^-$	311.0403	311.0421	1.79	**135.0447(100)**, 149.0089, **179.0352**, 134.0372	**0.27**
**11**	4.17	**Fertaric acid**	$C_{14}H_{13}O_9^-$	325.056	325.0600	4.04	**134.0368(100)**, **193.0506**, 178.027, 149.0089	**1.4**
**12**	7.81	**Aesculin**	$C_{15}H_{15}O_9^-$	339.0716	339.0734	1.79	**161.0241(100)**, 159.0295, 133.0285, **177.0398**, 115.0392	–
**13**	3.84	**Caffeoylquinic acid (like Chlorogenic acid)**	$C_{16}H_{17}O_9^-$	353.0873	353.0887	1.44	**191.0559(100)**, 161.0239, 127.0395, 173.0451, **135.0449**	–
				Flavan-3-ols and procyanidins				
**14**	3.42	**Catechin**	$C_{15}H_{13}O_6^-$	289.0712	289.0727	1.49	**123.045(100)**, 109.0294, 125.0244, 151.0398, 137.0244, 203.0712, 149.025, 221.0821, 187.0402, 245.0813	**1.4**
**15**	6.13	**Epicatechin**	$C_{15}H_{13}O_6^-$	289.0712	289.0727	1.49	**123.045(100)**, 109.0294, 125.0244, 151.0399, 137.0243, 203.0713, 149.0253, 221.0819, 187.0403, 245.0820	**2.5**
**16**	2.48	**Procyanidin B type dimer is. I**	$C_{30}H_{25}O_{12}^-$	577.1346	577.1365	1.90	**289.0724(100)**, **407.0780**, 125.0243, 245.0805, 161.0248, 137.0242, 273.0408, **425.0884**, **451.1036**, 255.0339, 229.0511	**2.9**
**17**	4.11	**Procyanidin B type dimer is. II**	$C_{30}H_{25}O_{12}^-$	577.1346	577.1365	1.90	**289.0718(100)**, **407.0776**, 125.0241, 245.0798, 161.0249, 137.0239, 273.0404, **425.0885**, **451.1047**, 255.0377, 229.0512, 205.0485	–

**18**	5.38	**Procyanidin B type dimer is. III**	$C_{30}H_{25}O_{12}^{-}$	577.1346	577.1365	1.90	**289.0722(100)**, **407.0778**, 125.0242, 245.0803, 161.0250, 137.0242, 273.0407, **425.0882**, **451.1031**, 229.0512, 205.0476, 109.0291	**2.0**
**19**	3.41	**Chalcan-flavan 3-ol dimer is. I (like Gambiriin A1)**	$C_{30}H_{27}O_{12}^{-}$	579.1503	579.1522	1.95	**289.0720(100)**, 245.0824, 271.0607, 179.0352, 205.0510, 165.0187, 151.0400, 137.0245, 125.0242, 109.0293	–
**20**	6.07	**Chalcan-flavan 3-ol dimer is. II**	$C_{30}H_{27}O_{12}^{-}$	579.1503	579.1522	1.95	**289.0719(100)**, 245.0824, 271.060719, 179.0352, 205.0510, 165.0188, 151.0397, 137.0241, 125.0241, 109.0293, 221.0825	–
**21**	6.84	**Procyanidin dimer B type gallate**	$C_{37}H_{29}O_{16}^{-}$	729.1456	729.1481	2.54	**407.0772(100)**, **289.0716**, 125.0239, 451.1023, **169.0141**, 577.1319, 271.0612, 287.0567, 441.0825, 161.0246, 245.0591, 203.0206	–
				Flavonols and glycosides				
**22**	10.1	**Kaempferol**	$C_{15}H_{9}O_{6}^{-}$	285.0399	285.0411	1.19	**285.0405(100)**, 185.0609, 229.0515, 239.035, 159.0447, 211.0396, 143.0497, 151.0038, 227.0347, 255.0301, 268.0370	–
**23**	9.30	**Quercetin**	$C_{15}H_{9}O_{7}^{-}$	301.0348	301.0368	1.97	**151.0036(100)**, 121.0292, **178.9984**, 149.0237, 301.0334, 245.0456, 273.0400, 229.0500, 201.0549	**3.5**
**24**	10.3	**Isorhamnetin**	$C_{16}H_{11}O_{7}^{-}$	315.0505	315.0516	1.12	**300.0276(100)**, 151.0033, 301.031, 107.0133, 271.0251, 283.0259, 255.0293, 227.0344, 243.0301, 179.0001	**8.6**
**25**	8.41	**Myricetin**	$C_{15}H_{9}O_{8}^{-}$	317.0297	317.0315	1.76	**151.0036(100)**, 137.0241, 107.0137, 178.9987, 165.0191, 227.0349, 243.0311, 271.0247, 317.0306	**1.3**
**26**	9.27	**Laricitrin**	$C_{16}H_{11}O_{8}^{-}$	331.0454	331.0473	1.91	**151.0062(100)**, 316.0231, 178.9995, 271.0243, **317.0257**, 287.0179, 259.0252, 243.0300, 107.0135	–
**27**	7.72	**Syringetin**	$C_{17}H_{13}O_{8}^{-}$	345.061	345.0634	2.36	**190.9994(100)**, **315.0144**, 163.0028, 287.0211, **330.0383**, 316.019, 271.0243, 259.0244, 243.0282, 345.0607	–
**28**	7.60	**Quercetin 3-*O*-hexuronide**	$C_{21}H_{17}O_{13}^{-}$	477.0669	477.0687	1.78	**301.0358(100)**, 151.0034, 178.9984, 283.0251, 273.0403, 255.0301, 245.0451	**2.1**
**29**	7.13	**Myricetin 3-*O*-hexoside**	$C_{21}H_{19}O_{13}^{-}$	479.0826	479.0847	2.13	**316.0229(100)**, 271.0245, 287.0194, 178.9982, 151.0035, 479.0832	**11.9**
**30**	7.05	**Myricetin 3-*O*-hexuronide**	$C_{21}H_{17}O_{14}^{-}$	493.0618	493.0647	2.87	**317.0304(100)**, 318.0312, 178.9971, 151.0049, 137.0232, 271.0281, 299.0174	–
**31**	7.65	**Laricitrin 3-*O*-hexoside**	$C_{22}H_{21}O_{13}^{-}$	493.0982	493.0988	0.58	**330.0382(100)**, 331.0446, **315.0150**, 316.0201, 287.02, 493.1013, 271.0245, 243.0285, 151.0055, 178.9975	–

**32**	8.11	**Syringetin 3-*O*-hexoside**	$C_{23}H_{23}O_{13}^{-}$	507.1139	507.1156	1.73	**344.0541(100)**, 345.0591, 507.1147, 273.0405, 301.0369, **316.0588**, **329.0321**, 258.0160, 151.0034	**1.7**
		Other detected non-anthocyanin flavonoids						
**33**	9.83	**Naringenin**	$C_{15}H_{11}O_{5}^{-}$	271.0606	271.0622	1.55	**119.0501(100)**, 151.0034, 107.0133, 177.0182, 161.0586, 145.0275, 229.0541	**1.4**
**34**	7.39	**Taxifolin**	$C_{15}H_{11}O_{7}^{-}$	303.0505	303.0522	1.72	**125.0249(100)**, 151.0216, 174.0312, 199.0390, 137.0211, 193.0515, 243.0271	–
**35**	5.05	**Dihydromyricetin**	$C_{15}H_{11}O_{8}^{-}$	319.0454	319.0469	1.51	**125.0242(100)**, 165.019, 151.0038, 167.0346, 137.0241, 175.0040, 193.0137, 205.0501, 233.0457	–
**36**	8.40	**Phloretin 2′-*O*-hexoside (like Phlorizin)**	$C_{21}H_{23}O_{10}^{-}$	435.1291	435.1316	2.48	**167.0351(100)**, **273.0778**, 125.0238, 274.0802, 179.0348, 123.0452, 168.0388	–
		Stilbenoids						
**37**	9.34	**Resveratrol**	$C_{14}H_{11}O_{3}^{-}$	227.0708	227.0721	1.28	**143.0501(100)**, 185.0593, 117.0347, 157.0655, 167.0535	**2.0**
**38**	8.22	**Resveratrol hexoside (like Piceid)**	$C_{20}H_{21}O_{8}^{-}$	389.1236	389.1253	1.66	**227.0711(100)**, 185.0605, 143.0499, 159.0811	**1.1**
		Anthocyanins and pyranoanthocyanins						
		Malvidin derivatives						
**39**	6.59	**Malvidin 3-*O*-glucoside**	$C_{23}H_{25}O_{12}^{+}$	493.1346	493.1375	2.9	**331.0831(100)**, 332.0854, 315.0508, 316.0578, 287.0555	**8.0**
**40**	7.13	**Malvidin 3-*O*-hexoside-acetaldehyde (Vitisin B)**	$C_{25}H_{25}O_{12}^{+}$	517.1346	517.1367	2.1	**355.0819(100)**, 356.0854, 317.0662	**3.4**
**41**	7.40	**Malvidin 3-*O*-(6"-acetyl)hexoside**	$C_{25}H_{27}O_{13}^{+}$	535.1452	535.1475	2.33	**331.0819(100)**, 332.085, 333.0878, 315.0505	–
**42**	7.45	**10H-Pyranomalvidin 3-*O*-(6"-acetyl)hexoside (Malvidin-acetaldehyde adduct I)**	$C_{27}H_{27}O_{13}^{+}$	559.1452	559.147	1.83	**355.0822(100)**, 356.0848, 397.0921	–
**43**	7.12	**Malvidin 3-*O*-hexoside-pyruvate (Vitisin A)**	$C_{26}H_{25}O_{14}^{+}$	561.1244	561.1266	2.17	**399.0722(100)**, 400.0754	**1.7**
**44**	8.64	**Malvidin 3-*O*-hexoside-4-vinylphenol**	$C_{31}H_{29}O_{13}^{+}$	609.1608	609.1626	1.78	**447.1079(100)**, 448.1112, 431.0755	–
**45**	8.39	**Malvidin 3-*O*-hexoside-4-vinylcatechol (Pinotin A)**	$C_{31}H_{29}O_{14}^{+}$	625.1557	625.1577	1.97	**463.1026(100)**, 464.1059, 447.0745	–
**46**	8.22	**Malvidin 3-*O*-(6″-*p*-coumaroyl)hexoside**	$C_{32}H_{31}O_{14}^{+}$	639.1714	639.1739	2.52	**331.0819(100)**, 332.085, 333.0876	–
**47**	8.11	**10H-Pyranomalvidin 3-*O*-(6"-*p*-coumaroyl) hexoside (Malvidin-acetaldehyde adduct II)**	$C_{34}H_{31}O_{14}^{+}$	663.1714	663.1737	2.32	**355.0811(100)**, 356.0852, 357.087	–
		Other detected anthocyanins						
**48**	6.06	**Petunidin 3-*O*-glucoside**	$C_{22}H_{23}O_{12}^{+}$	479.119	479.1205	1.55	**317.0657(100)**, 318.0698, 302.0423	–

**49**	7.59	**Peonidin 3-*O*-(6"- acetyl)hexoside**	$C_{24}H_{25}O_{12}^{+}$	505.1346	505.1362	1.6	**301.0704(100)**, 302.0746, 286.048	–
**50**	8.30	**Peonidin 3-*O*-(6″-*p*-coumaroyl)hexoside**	$C_{31}H_{29}O_{13}^{+}$	609.1608	609.1635	2.68	**301.0708(100)**, 302.0744, 303.076, 286.0477	–
**51**	8.06	**Petunidin 3-*O*-(6″-*p*-coumaroyl)hexoside**	$C_{31}H_{29}O_{14}^{+}$	625.1557	625.1581	2.37	**317.0661(100)**, 318.0689, 302.0466	–

* **Abbreviations:** is.-isomers; “–“ compounds identified only in young or aged wine; **YPW**-Young Prokupac wine; **APW**-Aging Prokupac wine; Ratio of each compound identified in young and aged wine, calculated as ratio of their areas (**Ratio YPW/APW**).

Table S3. Polypetide composition (%) of saliva proteins

No. Saliva Band	LMW (kDa)	CSP (%)
4	61.9	4.02
7	46.8	4.49
8	44.5	4.06
9	35.4	5.40
10	33.2	9.25
12	29.4	13.42
14	25.3	7.28
15	19.1	7.50
17	17.4	7.67
**∑ 9-17 (PRPs)**		**50.52**
19	13.1	6.45
20	11.7	8.52
21	10.9	8.06
**∑ 19-21 (Cystatins)**		**23.02**
23	6.9	7.98
24	5.5	5.90
**∑ 23-24 (Statherins)**		**13.88**
-	-	**100**

Table S4. One-way ANOVA for sensory parameters "acidity," "bitterness," "astringency," and "tannin quality" (factors wine samples and panelists).

Sensory parameter	*SS*	*MS*	*F*	*p-value*	*SS*	*MS*	*F*	*p-value*
	Wine samples				*Panelists*			
Acidity	0.9112	0.9112	5.1298	0.0337	1.8920	0.1720	0.7051	0.7148
Bitterness	0.0430	0.04230	0.3810	**0.5434**	0.5386	0.0490	0.2960	0.9736
Astringency	0.7950	0.7950	5.8130	0.0247	1.1859	0.1078	0.4941	0.8734
Tannin Quality	2.5203	2.5203	35.1272	$5.7816x10^{-6}$	0.7892	0.0717	0.2602	0.9834

Abbreviations: *SS* – Sum of Squares; *MS* – Mean of Squares; *F* – F-statistics.

Table S5. Results of sensory analysis of wines by *Buxbaum* method

Sensory parameter	*Wine samples*	
	YPW	*APW*
Colour	2.0	2.0
Clearness	2.0	2.0
Aroma	3.2	3.6
Taste	10.4	10.4
**Total**	**17.6**	**18.0**
